# Design and validation of a semi-automatic bone segmentation algorithm from MRI to improve research efficiency

**DOI:** 10.1038/s41598-022-11785-6

**Published:** 2022-05-12

**Authors:** Lauren N. Heckelman, Brian J. Soher, Charles E. Spritzer, Brian D. Lewis, Louis E. DeFrate

**Affiliations:** 1 Department of Orthopaedic Surgery, Duke University School of Medicine, DUMC Box 3093, Durham, NC 27710 USA; 2 Department of Biomedical Engineering, Pratt School of Engineering, Duke University, Durham, NC USA; 3 Department of Radiology, Duke University School of Medicine, Durham, NC USA; 4 Department of Mechanical Engineering & Materials Science, Pratt School of Engineering, Duke University, Durham, NC USA

**Keywords:** Biomedical engineering, Bone, Orthopaedics, Bone imaging, Magnetic resonance imaging, Tomography

## Abstract

Segmentation of medical images into different tissue types is essential for many advancements in orthopaedic research; however, manual segmentation techniques can be time- and cost-prohibitive. The purpose of this work was to develop a semi-automatic segmentation algorithm that leverages gradients in spatial intensity to isolate the patella bone from magnetic resonance (MR) images of the knee that does not require a training set. The developed algorithm was validated in a sample of four human participants (in vivo) and three porcine stifle joints (ex vivo) using both magnetic resonance imaging (MRI) and computed tomography (CT). We assessed the repeatability (expressed as mean ± standard deviation) of the semi-automatic segmentation technique on: (1) the same MRI scan twice (Dice similarity coefficient = 0.988 ± 0.002; surface distance = − 0.01 ± 0.001 mm), (2) the scan/re-scan repeatability of the segmentation technique (surface distance = − 0.02 ± 0.03 mm), (3) how the semi-automatic segmentation technique compared to manual MRI segmentation (surface distance = − 0.02 ± 0.08 mm), and (4) how the semi-automatic segmentation technique compared when applied to both MRI and CT images of the same specimens (surface distance = − 0.02 ± 0.06 mm). Mean surface distances perpendicular to the cartilage surface were computed between pairs of patellar bone models. Critically, the semi-automatic segmentation algorithm developed in this work reduced segmentation time by approximately 75%. This method is promising for improving research throughput and potentially for use in generating training data for deep learning algorithms.

## Introduction

Recent advances in orthopaedic research have relied heavily on medical imaging to investigate in vivo biomechanics^1–7^. In particular, previous studies have used magnetic resonance imaging (MRI)-based, three-dimensional (3D) solid modeling techniques to investigate in vivo exercise-induced cartilage deformations^8–11^, ligament/tendon elongations^12,13^, and knee kinematics^14–17^, to name a few. These techniques often require researchers to manually segment bones from magnetic resonance (MR) images, which is a laborious process. The automation of bone segmentation has the potential to greatly improve research efficiency and throughput.

Manual segmentation of bones from MR images takes many hours per MR scan. As a result, this process can be cost- and time-prohibitive. Because there is a need for ways to reduce analysis time, some groups have used semi-automated algorithms^18–22^, specialized MRI pulse sequences with computed tomography (CT)-like bone contrast^3,23–29^, statistical shape modeling ^4^, interactive image segmentation^30,31^, and deep learning^5,6^ to improve the efficiency of the segmentation process.

Although MRI is regarded for its soft-tissue visualization, bone signal in most MR images is low as compared to other imaging modalities such as radiography and CT. Most boundary detection algorithms rely on gradients in signal intensity (including Prewitt^32^, Marr-Hildreth^33^, Canny^34^, and Sobel^35^). Therefore, contrast is a critical feature for segmentation. Specifically, a high contrast (or a large intensity gradient) between bone and the surrounding soft tissue is critical for boundary detection algorithms to be able to isolate bones. Contrast between the bone and cartilage tissue can be quantified using the following Eq.^36^:

1 $$Contrast=\left|\frac{Intensit{y}_{Bone}-Intensit{y}_{Cartilage}}{Intensit{y}_{Cartilage}}\right|$$

Larger contrast values indicate greater signal differences between these adjacent tissues.

The purpose of this investigation was to develop and validate a semi-automatic boundary detection algorithm to isolate the bones from MR images. We hypothesized that our semi-automatic bone segmentation algorithm would provide a comparable accuracy to both manual segmentation of MR images and the semi-automatic segmentation of CT images, especially near bone regions adjacent to articular cartilage.

## Methods

**Figure 1:**
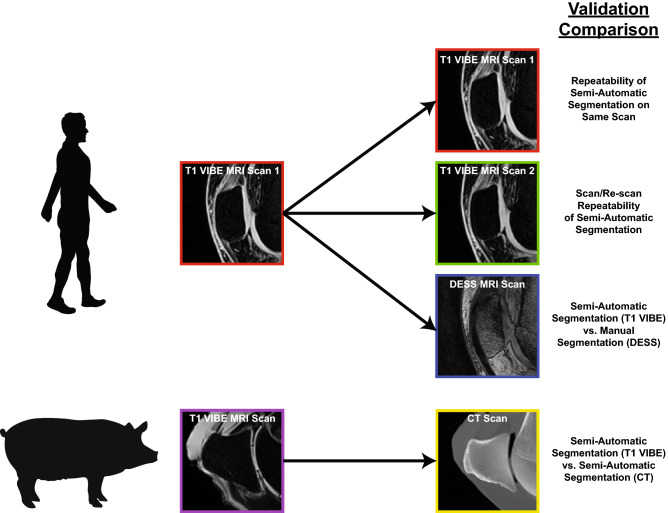
Overview of comparisons made between segmentation techniques. In human participants, the semi-automatic segmentations of T1 VIBE MRI scans were compared to: (1) repeated semi-automatic segmentations of the same T1 VIBE MRI scans, (2) semi-automatic segmentations of second T1 VIBE MRI scans of the same participants, and (3) manual segmentations of DESS MRI scans of the same participants. In porcine specimens, the semi-automatic segmentations of T1 VIBE MRI scans were compared to the semi-automatic segmentations of CT scans of the same specimens. *T1 VIBE* T1-weighted Volume-Interpolated Breathhold Examination with Water Excitation, *DESS* Double Echo Steady-State, *MRI* Magnetic Resonance Imaging, *CT* Computed Tomography.

We performed four unique segmentation comparisons to validate our semi-automatic segmentation algorithm (Fig. 1). First, we compared the repeatability of our proposed technique by applying the semi-automatic bone segmentation algorithm to the same MRI scan twice. Second, we assessed how well our semi-automatic bone segmentation algorithm could isolate the patella from two different MRI scans of the same human participants. Third, we evaluated the differences between manual and semi-automatic segmentations of MRI scans of the same human participants. Finally, we compared the semi-automatic segmentations of MRI and CT scans of the same porcine specimens. We imaged porcine specimens to avoid subjecting our human volunteers to the ionizing radiation present during CT imaging. Additional information regarding the in vivo and ex vivo imaging protocols and the segmentation validation methods will be described in more detail below.

### Recruitment & in vivo imaging protocol

**Figure 2:**
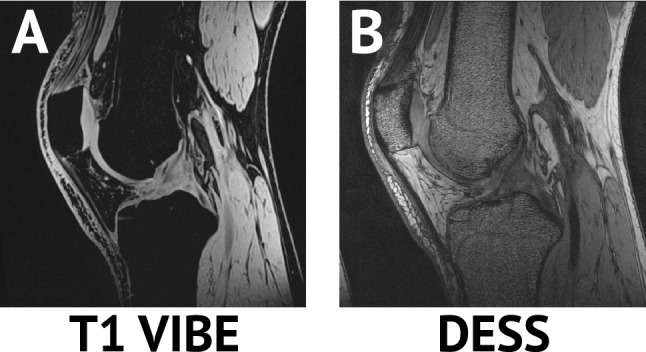
Sagittal (**A**) T1 VIBE and (**B**) DESS MR images were acquired of each human participant’s dominant knee. While DESS images have been reliably used for knee joint bone and cartilage model generation in the past, T1 VIBE had greater contrast between the bone and adjacent cartilage (0.95 vs. 0.72). *DESS* Double Echo Steady-State, *T1 VIBE* T1-weighted Volume-Interpolated Breathhold Examination with Water Excitation.

**Figure 3:**
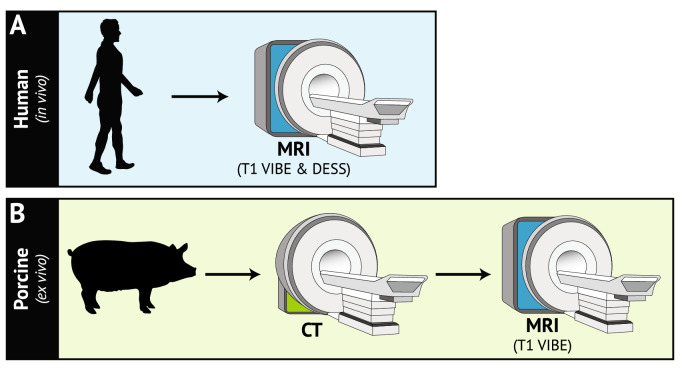
Imaging protocols for the in vivo and ex vivo arms of the experiment. (**A**) T1 VIBE and DESS MR images were acquired of each human (in vivo) participant’s dominant knee. (**B**) The porcine (ex vivo) imaging protocol consisted of CT imaging followed by the same T1 VIBE MRI sequence performed on the human participants. *T1 VIBE* T1-weighted Volume-Interpolated Breathhold Examination with Water Excitation, *DESS* Double Echo Steady-State, *MR* magnetic resonance, *CT* computed tomography.

Four healthy participants (1 male, 3 females; mean age: 28 years, range 23–38 years; mean body mass index (BMI): 22.8 kg/m^2^, range 21.5–24.2 kg/m^2^) were recruited and enrolled in this Duke University Institutional Review Board-approved study. All protocols adhered to the approved guidelines, and informed consent was received from all subjects prior to participation in the investigation. Exclusion criteria included any history of lower-extremity injury, surgery, or pain prior to the study. All individuals underwent MR imaging of their dominant knee (defined as the leg with which they would kick a ball^37^) at 7AM in the Center for Advanced Magnetic Resonance Development within the Duke University Hospital on a 3.0 T MR scanner (TIM Trio; Siemens Healthcare; Malvern, PA) with an 8-channel knee coil (Invivo; Gainesville, FL). Due to previous work comparing bones segmented from CT images and various pulse sequences^38^, sagittal T1-weighted volume-interpolated breathhold examination with water excitation (T1 VIBE) MR images were acquired (Table 1; Figs. 2A; 3A). Double echo steady-state (DESS) MR images were also acquired for validation purposes, as these images have been used previously for manual segmentation of the patella (Table 1; Figs. 2B, 3A)^8,39,40^.

**Table 1:** MRI parameters.

Pulse sequence	T1 VIBE	DESS
Flip angle	10°	25°
Repetition time, TR (ms)	12	17
Echo time, TE (ms)	4.9	6
Matrix size (pixels)	248 $\times$ 256, interpolated to 496 $\times$ 512	512 $\times$ 512
Resolution (mm)	0.7 $\times$ 0.7 $\times$ 0.7, interpolated to 0.4 $\times$ 0.4 $\times$ 0.7	0.3 $\times$ 0.3 $\times$ 1
Acquisition time (minutes:seconds)	4:28	9:49
Water excitation	Yes	No
Contrast	0.95	0.72
Rationale for testing	Agreement with CT^38^	Knee^8,39^, Ankle^41^

*T1 VIBE* T1-weighted volume-interpolated breathhold examination with water excitation, *DESS* double echo steady-state.

### Porcine stifle joint preparation & ex vivo imaging protocol

Three intact porcine stifles were obtained from a local abattoir. The joints were stored in a cold room at 4 °C prior to CT imaging in the Duke Cancer Center (Fig. 3B; scanner: Somatom Definition Flash, Siemens Healthcare; Malvern, PA; matrix: 512 × 512 pixels; resolution: 0.3 × 0.3 × 0.6 mm; voltage potential (peak): 120 kVp; tube current: 240 mA). Next, all joints were stored overnight in the 4 °C cold room and then imaged the following day in the Duke Center for Advanced Magnetic Resonance Development using the same T1 VIBE MRI sequence as was used for the human in vivo scans (Table 1; Fig. 3B).

### Semi-automatic bone segmentation algorithm

**Figure 4:**
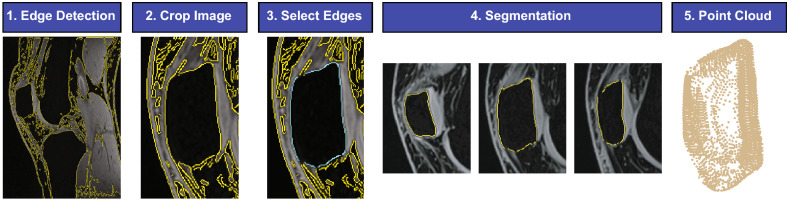
Semi-automatic segmentation steps for isolating bone from either MR or CT images. (**1**) A Canny edge detection filter is used to identify all edges in each image. The edges are overlaid in yellow on each image. (**2**) The image volume is cropped around the bone of interest (patella). (**3**) On a single starting image slice, the user selects the edges of the patella previously identified by the Canny filter. The selected edges are highlighted in cyan. (**4**) This starting slice is then used to determine the edges nearest these points in adjacent slices in the image volume. The user can remove stray edge points in any slice before proceeding. (**5**) The identified bone edges are converted into a three-dimensional point cloud.

We developed an algorithm to semi-automatically segment the bones from both MR and CT images in MATLAB (The MathWorks, Inc.; Natick, MA). The major steps of this process are outlined in Fig. 4 and will be further explained in the subsequent sections.

A custom MATLAB graphical user interface (GUI) was designed to carry out the segmentation steps. To use this program, T1 VIBE MR images (human and porcine) or sagittal CT images (porcine only) are imported into MATLAB.

The edges in each image are identified using a Canny filter in MATLAB (Fig. 4, Step 1)^34^. The threshold and standard deviation of the Canny filter are manually selected to maximize the identification of the bone edges, while also limiting noise. The threshold of the Canny filter is defined using a value on a normalized scale from 0–1. All edges stronger than this threshold are preserved. We aimed to keep our threshold as low as possible (to preserve as many edges as possible), while still removing some extraneous edges due to image noise. The selected thresholds were approximately equal for all scans within a given modality (range 0.02–0.07 for MRI, 0.15–0.25 for CT), and the default standard deviation (0.10) was used for all scans. Next, the first and last slices containing the bone of interest are manually identified. The image stack is then manually cropped to isolate the bone of interest (Fig. 4, Step 2). Next, the user selects slice(s) to initialize using a multi-step process (Fig. 4, Step 3):

1. Use drop-down menu to select a slice to initialize.
2. Remove points to break the 8-pixel connectivity (up, down, left, right, and four corners) of Canny edges that are not along the bone boundary.
3. Select edges around the bone boundary to keep.
4. Repeat steps 1–3 until all chosen slices are initialized.

**Figure 5:**
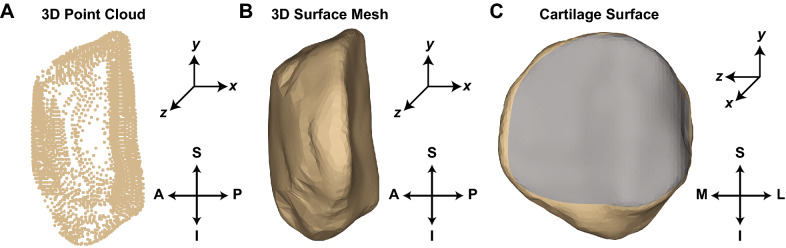
(**A**) Lateral view of a 3D point cloud model generated from semi-automatic segmentations of a human patella. (**B**) Lateral view of a 3D surface mesh generated from the 3D point cloud in (**A**). (**C**) Posterior view of the 3D surface mesh in (**B**). The gray region depicts the area in which cartilage is located. *A* anterior, *P* posterior, *S* superior, *I* inferior, *M* medial, *L* lateral).

The bone edges from each initialized slice are used as a starting point from which to extract the nearest Canny edge pixels from the image immediately before and after the chosen slice. In this study, the edges were initialized in approximately one out of every five images. Following slice initialization, the user can step through the remaining images one at a time so corrections can be made before proceeding to the next image (Fig. 4, Step 4). After all images are processed, the user can export the coordinates of a 3D point cloud of the designated bone (Fig. 4, Step 5; Fig. 5A).

### Validation—Dice similarity coefficient

To determine the repeatability of the semi-automatic segmentation algorithm, we applied this technique to the T1 VIBE MR scans of the human patellae twice per scan. This enabled us to determine the Dice similarity coefficient (DSC) of our segmentation outcomes^42^:

2 $$DSC=\frac{2\left|X\cap Y\right|}{\left|X\right|+\left|Y\right|}$$

where $\left|X\cap Y\right|$ represents the number of elements the sets $X$ and $Y$ have in common, and $\left|X\right|$ and $\left|Y\right|$ represent the number of elements in the sets $X$ and $Y$, respectively. Dice similarity coefficients range from 0 to 1, where 0 indicates no agreement between datasets and 1 indicates full agreement between datasets. We computed the DSC for each MR slice containing patellar bone and then averaged the coefficients across all slices (weighted based on how many pixels were present in each slice) for a given participant to yield an overall mean value per subject. We used Dice similarity coefficients for this comparison since the semi-automated algorithm was repeated twice on the same images.

### Validation—surface distance

To further validate the semi-automatic segmentation algorithm, we quantified the mean surface distance between bone models obtained using different segmentation techniques^43^. Specifically, we focused on analyzing the bone-cartilage interfaces, as this is critical for assessing changes in cartilage thickness^8,39,40,44^. We first assessed the repeatability of the semi-automatic segmentation of T1 VIBE MR images of human patellae by isolating the patella from: (1) the same image volume twice and (2) repeated T1 VIBE MR scans of the same human patellae acquired approximately 30 min apart on the same day (scan/re-scan repeatability). We also compared bone models generated from: (1) the semi-automatic segmentation of T1 VIBE MR images of human patellae to the manual segmentation of DESS MR images of the same human patellae^8–10,40,45^ and (2) the semi-automatic segmentation of T1 VIBE MR images of porcine patellae to the semi-automatic segmentation of CT images of the same porcine patellae.

To assess the surface distance between pairs of bones, 3D point clouds of the patellae from each of the four human participants (MRI only) and the three porcine specimens (MRI & CT) were generated using the semi-automatic bone segmentation algorithm just described (Fig. 5A). The four human patellae were also manually segmented from each DESS MR image using solid modeling software (Rhinoceros; Robert McNeel and Associates; Seattle, WA) to form 3D point clouds^8,39,40,44^. Next, the MRI and CT bone point clouds were imported into Geomagic Studio (Geomagic, Inc.; Cary, NC), and the porcine CT point clouds were registered to their respective MRI point clouds using an iterative closest point algorithm to ensure site-specific comparisons of the bone surfaces. A similar registration process was used to align the semi-automatic and manual point clouds of the human patellae, as well as the point clouds generated from the semi-automatic segmentation of the repeated (scan/re-scan) T1 VIBE MR acquisitions in the human participants.

Following registration, the bone models were converted into 3D surface meshes (Fig. 5B). The patellar bone surface directly in contact with patellar cartilage was also extracted from each 3D model (Fig. 5C).

**Figure 6:**
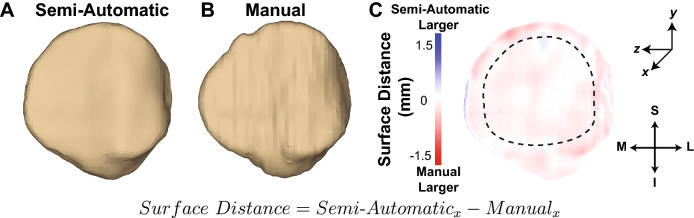
Posterior views of representative 3D bone models of a human patella generated using (**A**) semi-automatic segmentation of sagittal T1 VIBE MR images and (**B**) manual segmentation of sagittal DESS MR images. The same post-processing and smoothing operators were applied to both models. The DESS images had a larger slice thickness as compared to the T1 VIBE images (1 mm vs. 0.7 mm), which may contribute to differences in smoothness between the models. (**C**) The surface distances map shows strong agreement between the 3D models. Blue indicates regions where the semi-automatic segmentation model was larger than the manual segmentation model, whereas red indicates regions where the manual segmentation model was larger than the semi-automatic segmentation model. The black dashed line represents the bone region analyzed after the 25% perimeter reduction of the cartilage boundary, which was implemented to minimize edge effects. Surface distance was defined as the difference between the x-coordinates of the two bone models. *T1 VIBE* T1-weighted Volume-Interpolated Breathhold Examination with Water Excitation, *DESS* Double Echo Steady-State, *MR* magnetic resonance, *S* superior, *I* inferior, *M* medial, *L* lateral).

To quantitatively compare the agreement between two bone surface meshes, pairs of surface meshes were evaluated using MATLAB (Fig. 6A,B). While the same process was performed for each pair of surface meshes to quantify the resulting surface distances, we will describe the semi-automatic vs. manual MRI segmentation (T1 VIBE and DESS MR images, respectively) evaluation below.

The semi-automatic segmentation bone model (from T1 VIBE MR images), the manual segmentation bone model (from DESS MR images), and the bone model mesh faces located adjacent to articular cartilage in both models were imported into MATLAB, and the volume centroids of the semi-automatic segmentation bone model were subtracted from all three models to center them about the origin while maintaining their registration. Next, a search algorithm was employed to find all mesh nodes on the manual segmentation bone model located within a 2.5 mm radius of each mesh node on the semi-automatic segmentation bone model. These mesh node coordinates were averaged to yield a single vertex. This yielded a set of matched vertices on the two bone models from which to directly compute the surface distance. Surface distance was defined as the difference in the x-coordinates between the two bone models, as the x-direction is perpendicular to the articular surface of the patella (Fig. 6). Positive surface distances were indicative of regions where the semi-automatic segmentation model was larger than the manual segmentation model, whereas negative surface distances were indicative of regions where the manual segmentation model was larger than the semi-automatic segmentation model.

While we are generating whole-bone models, it is important to note that we are primarily concerned with the agreement between the regions adjacent to the articular surfaces of the bone models since these regions are the areas that will influence cartilage thickness measurements^8,39,40,44^. Thus, the surface distance calculations were further refined by extracting only bone mesh nodes within the confines of the patellar cartilage model. Specifically, only bone mesh nodes located adjacent to patellar cartilage after a 25% perimeter reduction of the cartilage boundary were considered (Fig. 6C). A similar perimeter reduction was implemented previously to avoid edge effects when quantifying running-induced patellar cartilage strains continuously across the articular surface^39^. All other surface distance comparisons (repeated segmentations of the same T1 VIBE MRI scans, scan/re-scan repeatability of T1 VIBE MRI segmentations of the same participants, and T1 VIBE MRI vs. CT segmentations of the same porcine specimens) were performed using the same methodology.

## Results

A single experienced investigator required approximately 15 min to segment the patella bone from T1 VIBE MR images using the semi-automatic bone segmentation algorithm and approximately 1 h to manually segment the patella bone from the DESS MR images, yielding a time savings of about 75%. The mean (± standard deviation) Dice similarity coefficient and surface distance of our repeated semi-automatic segmentations on the same T1 VIBE MRI scans were 0.988 ± 0.002 and − 0.01 ± 0.001 mm, respectively, indicating a high level of repeatability in isolating the patellar bone from T1 VIBE MR images (Table 2). The negative mean surface distance indicates that the second segmentation of the patella was slightly larger than the first, on average. The semi-automatic T1 VIBE MRI segmentation algorithm yielded a mean (± standard deviation) surface distance of − 0.02 ± 0.08 mm as compared to manual DESS MRI segmentations in the human participants, indicating the DESS MRI segmentations were larger than the semi-automatic MRI segmentations (Table 2). Similarly, when segmenting the same individual’s patella twice (scan/re-scan on the same day), the semi-automatic T1 VIBE MRI segmentation algorithm generated segmentations with a mean surface distance of − 0.02 ± 0.03 mm, indicating the second segmentation was, on average, 0.02 mm larger than the first (Table 2). Finally, the semi-automatic T1 VIBE MRI and CT segmentations of the porcine patellae resulted in a mean surface distance of − 0.02 ± 0.06, indicating the semi-automatic CT segmentations were larger than the semi-automatic T1 VIBE segmentations (Table 2).

**Table 2:** Surface Distance Results for Each Validation Comparison.

Validation Comparison	Species	Sample Size	x-Axis Surface Distance (mm)
Semi-automatic T1 VIBE MRI repeatability (same scan)	Human	4	− 0.01 ± 0.001
Semi-automatic T1 VIBE MRI repeatability (scan/re-scan)	Human	4	− 0.02 ± 0.03
Semi-automatic T1 VIBE MRI vs. manual DESS MRI	Human	4	− 0.02 ± 0.08
Semi-automatic T1 VIBE MRI vs. semi-automatic CT	Porcine	3	− 0.02 ± 0.06

Data presented as mean ± standard deviation. *T1 VIBE* T1-weighted volume-interpolated breathhold examination with water excitation, *DESS* double echo steady-state, *MRI* magnetic resonance imaging, *CT* computed tomography.

## Discussion

We have developed a semi-automatic segmentation algorithm to isolate bone from T1 VIBE MR images using gradients in signal intensity. T1 VIBE MR images were chosen for this application because they provide excellent contrast between bone and the adjacent articular cartilage. The algorithm developed is repeatable in its ability to detect patellar bone boundaries from the same MR scan twice and from scan/re-scan acquisitions of the same participants. It also performs favorably in comparison to manual segmentation of MR images, and it produces comparable results between MR and CT images. Furthermore, this semi-automatic segmentation technique generates complete 3D point clouds in a fraction of the time it takes to manually segment the same images. These results indicate that the semi-automatic segmentation algorithm outlined in the present work may be a viable alternative for manual segmentation.

The semi-automatic segmentation algorithm developed in the present work was repeatable in isolating the patellar bone from T1 VIBE MR images. We quantified a mean Dice similarity coefficient of 0.988 and a mean surface distance of − 0.01 mm when comparing the segmentation results from the same MR scan twice. We further demonstrated that segmentations generated from two separate T1 VIBE MR scans of the same participant differed by a mean surface distance of − 0.02 mm. The semi-automatic segmentation algorithm also compared well with manual segmentation of MR images, resulting in a mean surface distance of − 0.02 mm. These values agreed with previous repeatability studies which have demonstrated that manual segmentation of bone cortices and cartilage surfaces from DESS MR images is repeatable to within approximately 0.03 mm^44^. Additionally, while manual segmentation of the patellar bone from MR images takes about an hour to complete, our semi-automatic segmentation algorithm takes approximately 15 min per patella. Thus, the present findings suggest the semi-automatic segmentation algorithm described can produce comparable results to those obtained via manual segmentation in a fraction of the time.

Furthermore, the semi-automatic segmentation algorithm yielded sub-millimeter mean surface distances when comparing the T1 VIBE MRI and CT bone models (− 0.02 mm). Previous work by Neubert et al.^38^ demonstrated that the T1 VIBE MR pulse sequence had sub-millimeter absolute differences between T1 VIBE and CT bone models of the femur, tibia, fibula, and patella. While zero echo time (ZTE) MRI mimics the visual appearance of CT images^23–29^, a previous study comparing ZTE MRI and CT scans of the glenoid bone showed the scans also differed by sub-millimeter values^29^. Thus, we believe T1 VIBE-based semi-automatic segmentation is a viable option for bone segmentation from MR images.

While we were able to reduce segmentation time by approximately 75%, further work may seek to reduce this processing time even further. Advances in deep learning algorithms may be one approach. However, deep learning techniques traditionally require relatively large datasets to train the resulting algorithms^46–48^, and the algorithm is limited by the quality of the training data. Since the semi-automatic segmentation technique described in the present work was shown to be comparable to manual segmentation, this method can potentially be used to generate training data for future deep learning algorithms.

Although the present work only validated the semi-automatic segmentation of a relatively small group of patellae, the algorithm described herein can likely be implemented in T1 VIBE images of other bones. This is in part because of the gradient-based segmentation approach used, which may be applicable to new situations. This is potentially a strength of this technique compared to some deep learning techniques that are designed to perform feature identification or pattern recognition in a specific training set^49^. Image artifacts, due to things such as patient motion or the presence of metallic objects, may alter the efficacy of the present algorithm. Future work will further assess the robustness of the current approach.

The utility of the semi-automatic segmentation algorithm developed in this work is currently limited to bone isolation. As such, manual segmentation of the articular cartilage may be needed to quantify exercise-induced cartilage deformations in vivo^15,39,40^. In previous studies, bone and cartilage have both been manually segmented from DESS MR images of the knee^8,39,44^, as the DESS sequence provides an excellent compromise regarding contrast between bone and cartilage as well as between cartilage and synovial fluid. It may be necessary to acquire both T1 VIBE and DESS MR images (for semi-automatic bone segmentation and manual cartilage segmentation, respectively) to assess cartilage deformations in future studies. While acquiring both sequences would increase scan time (mm:ss; T1 VIBE = 4:28, DESS = 9:49, both = 14:17), the significant time savings during data analysis justify this added scan. While this study specifically utilized T1 VIBE MR images for the semi-automatic bone segmentation algorithm, any commercially available pulse sequence with a large contrast may be a viable option for gradient-based segmentation. Future investigations may seek to identify other suitable sequences for semi-automatic bone segmentation that can also be used for manual cartilage segmentation.

In conclusion, we developed a gradient-based semi-automatic bone segmentation algorithm that was repeatable and produced results comparable to both manual MRI segmentation and semi-automatic CT segmentation. We used a T1 VIBE MR pulse sequence, which provided excellent contrast between bone and the adjacent articular cartilage, enabling us to overcome the low cortical bone signal inherent in most MR images^23,24^. This newly developed algorithm reduced analysis time by approximately 75%. Thus, the semi-automatic bone segmentation algorithm is a viable replacement for manual segmentation that will improve research efficiency.
